# Noradrenaline and Adrenoreceptors Are Involved in the Regulation of Prostaglandin I2 Production in the Porcine Endometrium after Experimentally Induced Inflammation

**DOI:** 10.3390/ijms25126313

**Published:** 2024-06-07

**Authors:** Barbara Jana, Jarosław Całka, Aneta Andronowska, Aleksandra Mówińska, Krzysztof Witek, Katarzyna Palus

**Affiliations:** 1 Division of Reproductive Biology, Institute of Animal Reproduction and Food Research of the Polish Academy of Sciences, Tuwima 10, 10-748 Olsztyn, Poland; a.andronowska@pan.olsztyn.pl (A.A.); aleksandra.zlotkowska@uwm.edu.pl (A.M.); k.witek@pan.olsztyn.pl (K.W.); 2 Department of Clinical Physiology, Faculty of Veterinary Medicine, University of Warmia and Mazury, Oczapowskiego 13, 10-718 Olsztyn, Poland; calkaj@uwm.edu.pl (J.C.); katarzyna.palus@uwm.edu.pl (K.P.)

**Keywords:** endometrium, endometrial epithelial cells, inflammation, noradrenaline, adrenoreceptors, prostaglandin I2 formation and release

## Abstract

Endometritis is a common disease in animals, leading to disruption of reproductive processes and economic losses. Noradrenergic control of prostaglandin (PG)I2 formation by inflamed endometrium is unknown. We determined the involvement of α1-, α2- and β-adrenoreceptors (ARs) in noradrenaline-influenced PGI synthase (PGIS) protein abundance and PGI2 release from porcine (1) endometrial explants with *Escherichia coli* (*E. coli*)-induced inflammation in vivo, and (2) *E. coli* lipopolysaccharide (LPS)-treated endometrial epithelial cells. Experiment 1. *E. coli* suspension (*E. coli* group) or saline (CON group) was injected into the uterine horns. In both groups, noradrenaline increased endometrial PGIS abundance and PGI2 release versus the control values, and it was higher in the *E. coli* group than in the CON group. In the CON group, a noradrenaline stimulating effect on both parameters takes place through α1D-, α2C- and β2-ARs. In the *E. coli* group, noradrenaline increased PGIS abundance and PGI2 release via α1A-, α2(B,C)- and β(1,2)-ARs, and PGI2 release also by α2A-ARs. Experiment 2. LPS and noradrenaline augmented the examined parameters in endometrial epithelial cells versus the control value. In LPS-treated cells, β(1,2)-ARs mediate in noradrenaline excitatory action on PGIS protein abundance and PGI2 release. β3-ARs also contribute to PGI2 release. Under inflammatory conditions, noradrenaline via ARs increases PGI2 synthesis and release from the porcine endometrium, including epithelial cells. Our findings suggest that noradrenaline may indirectly affect processes regulated by PGI2 in the inflamed uterus.

## 1. Introduction

Inflammation of the uterus (endometritis, metritis) is a common disorder. Severe inflammation is the cause of disruption of reproductive processes and, consequently, a decrease in the profitability of production [1]. Uterine inflammation is found in animals predominantly after labour. It also develops following abortion, in females with anoestrus or who have not yet given birth, and after natural mating or insemination. Bacterial uterine infections, often caused by *Escherichia coli* (*E. coli*), frequently lead to the development of endometritis and metritis in animals and humans [2,3]. The occurrence of uterine inflammation depends on several factors, such as the host’s innate immune response, the species and number of microorganisms colonising the endometrium and uterine contractility [1]. Lipopolysaccharide (LPS) acts by the receptor complex consisting of two interacting receptors (CD14 and Toll-like receptor 4) and an associated protein (MD-2) expressed on the endometrial cells. Activation of this complex by LPS induces the transcription of genes related to the inflammatory process. The products of these genes lead to cellular alterations to eliminate invading pathogens [4,5,6,7]. Impairment of the endometrial immune defence mechanisms and/or myometrial contractile activity are the reasons for the formation, development and maintenance of inflammation [8,9].

Prostaglandins (PGs) are one of the mediators of inflammation. Little information is available on the synthesis of PGI2 in inflamed uteri. The contents of a stable metabolite of PGI2 (6-keto-PGF1α) were increased in the endometrium and myometrium, as well as in the washings from uteri of gilts with the *E. coli*-induced endometritis. These changes were accompanied by an elevation in PGI synthase (PGIS) [10]. PGI2 is a very important component in inflammatory reactions [11,12] and affects the contractility of the inflamed pig uterus [10]. The levels of 6-keto-PGF1α were higher in the peripheral blood of post-partum dairy cows with spontaneous uterine infections [13] and after intrauterine infusions of LPS [14].

The most numerous nerve fibres in the pig uterus are those of the sympathetic part of the peripheral nervous system [15]. The expression of α1(A,B,D)- and α2(A,B,C)-adrenoreceptor (AR) isoforms and β(1,2,3)-AR subtypes in the healthy uterus of pigs and other animal species has been reported [16,17,18,19,20,21,22,23]. Catecholamines are one of the factors influencing the production of PGE2 and PGF2α in healthy human [24], rat [25] and porcine [26,27] uteri.

Endometritis in pigs results in marked changes in uterine AR expression [23]. In the pig endometrium in an inflammatory state, noradrenaline (NA) and ARs play a role in PGE2 [27] and PGF2α [26] formation and secretion. The significance of ARs in the noradrenergic regulation of PGI2 synthesis in the inflammatory-changed uterus is still unknown. It has only been reported that under physiological conditions, NA and adrenaline increase this PG release by the human myometrium acting via α- and β-ARs [24]. It is hypothesised that through ARs, NA alters PGIS abundance and PGI2 concentration in porcine endometrial tissue and epithelial cells under inflammatory conditions. The determination of noradrenergic control of PGI2 formation in the inflamed endometrium may help to better understand the neuro–immuno–endocrine interactions, which play a role in the endometrial inflammatory process and function. Exploring this topic will markedly improve the outcomes of prophylactic and therapeutic management concerning uterine inflammation in domestic animals. In consideration of this, this study examined the involvement of α1, α2, and β ARs in the regulation of NA-affected PGIS protein abundance and PGI2 release from the inflamed porcine endometrium.

## 2. Results

### 2.1. Experiment 1.1: The Effect of NA and/or AR Antagonists on PGIS Protein Abundance in Endometrial Explants with E. coli-Induced Inflammation In Vivo

#### 2.1.1. AR Antagonists Alone

In the control (CON) group (pigs with injections of saline into the uterine horns) and the *E. coli* group (pigs with injections of *E. coli* suspension into the uterine horns), the PGIS protein abundance in response to all AR antagonists was lower (*p* < 0.001) than the NA action (Table 1). In the *E. coli* group, the enzyme abundance in response to α1A (*p* < 0.001), α1B (*p* < 0.01), α1D, α1ABD (*p* < 0.05), α2A (*p* < 0.05), α2B (*p* < 0.001), α2C (*p* < 0.01), α2ABC (*p* < 0.05), β1 (*p* < 0.01) and β3 (*p* < 0.001) antagonists was higher than in the CON group.

#### 2.1.2. NA Alone

**Figure 1:**
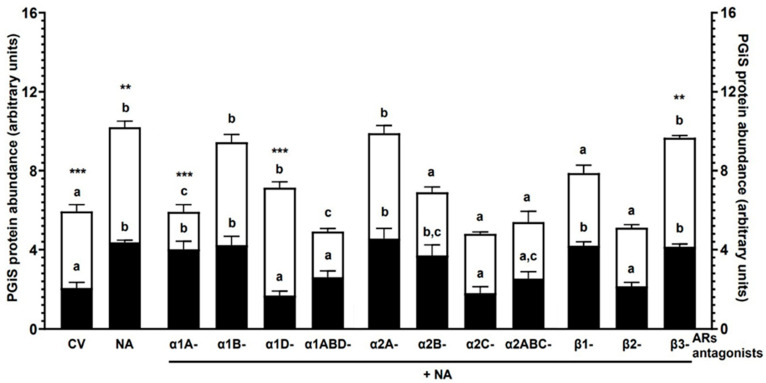
The influence of noradrenaline (NA, 10^−5^) alone or with antagonists of α1-, α2- and β-adrenoreceptors (ARs, 10^−4^ M) on the prostaglandin I synthase (PGIS) protein abundance in the endometrium of pigs of the CON (black bars) and *E. coli* (white bars) groups, determined by Western blotting. N = 5 for each group. Treatments were conducted in triplicate for each pig (n = 5) in the particular groups. PGIS values were normalised against glyceraldehyde-3-phosphate dehydrogenase (GAPDH) protein abundance. Data are presented as the mean ± sem. Representative bands for each group/treatment are presented on blots (Figure S1). Different letters (a, b, c) show statistical differences (*p* < 0.05–0.001) within each group for each subtype of α1- or α2-AR antagonists with NA or each subtype of β-AR antagonists with NA versus CV (control value, obtained from the CON or *E. coli* groups’ endometrial explants that had not undergone any treatment in vitro) and NA influence alone; means marked by the same letter within each group show no significant difference; ** *p* < 0.01, *** *p* < 0.001 show statistical differences between groups for the same treatment.

NA increased (*p* < 0.001) PGIS abundance in both groups in relation to the control values (obtained from the CON or *E. coli* groups’ endometrial explants that had not undergone any treatment in vitro) (Figure 1). The control value of the enzyme (*p* < 0.001) and that after using NA (*p* < 0.01) were higher in the *E. coli* group than in the CON group.

#### 2.1.3. α1-AR Antagonists with NA

PGIS protein abundance in the CON group after using α1A- and α1B-AR antagonists with NA was elevated (*p* < 0.001) compared to the control value (Figure 1). The α1D- (*p* < 0.001) and α1ABD (*p* < 0.01)-AR antagonists with NA decreased PGIS abundance in reference to the NA influence. In the *E. coli* group, α1A- and α1ABD-AR antagonists with NA reduced the enzyme abundance compared to the control value (*p* < 0.05) and NA action (*p* < 0.001). PGIS abundance in response to α1B- and α1D-AR antagonists with NA increased (*p* < 0.05) versus the control value. In relation to the CON group, the enzyme abundance in the *E. coli* group was higher (*p* < 0.001) after using α1A- and α1D-AR antagonists with NA.

#### 2.1.4. α2-AR Antagonists with NA

The α2A- (*p* < 0.001) and α2B (*p* < 0.05)-AR antagonists with NA increased the PGIS protein abundance in the CON group endometria in reference to the control value (Figure 1). Concerning the NA effect, the α2C- (*p* < 0.001) and α2ABC (*p* < 0.05)-AR antagonists with NA decreased this enzyme’s abundance. PGIS abundance in the *E. coli* group was elevated (*p* < 0.05) by the α2A-AR antagonist with NA compared to the control value. The α2B-, α2C- and α2ABC-AR antagonists with NA lowered (*p* < 0.001) the PGIS abundance versus the NA influence.

#### 2.1.5. β-AR Antagonists with NA

PGIS abundance was increased (*p* < 0.001) in the CON group tissue by β1- and β3-AR antagonists with NA versus the control value (Figure 1). The enzyme abundance was lowered (*p* < 0.001) by the β2-AR antagonist with NA versus the NA action. In the endometria of the *E. coli* group, β3-AR antagonist with NA elevated (*p* < 0.001) the PGIS abundance in relation to the control value. The enzyme abundance was decreased (*p* < 0.001) by β1- and β2-AR antagonists with NA compared to the NA influence. In the *E. coli* group, the PGIS abundance in response to the β3-AR antagonist with NA was higher (*p* < 0.01) than in the CON group.

The statistical comparison between the action of particular antagonists of α1- or α2-AR subtypes/isoforms with NA or particular antagonists of β-AR subtypes with NA and the action of NA on PGIS protein abundance is shown in Figure 1.

### 2.2. Experiment 1.2: The Effect of NA and/or AR Antagonists on the 6-keto-PGF1α Concentration in the Medium after Incubation of the Endometrial Explants with E. coli-Induced Inflammation In Vivo

#### 2.2.1. AR Antagonists Alone

In both groups, the 6-keto-PGF1α content in the medium after using all AR antagonists was lowered (*p* < 0.001) versus the NA action (Table 1). All AR antagonists in the *E. coli* group increased (*p* < 0.001) the content of this metabolite compared to the CON group.

#### 2.2.2. NA Alone

**Figure 2:**
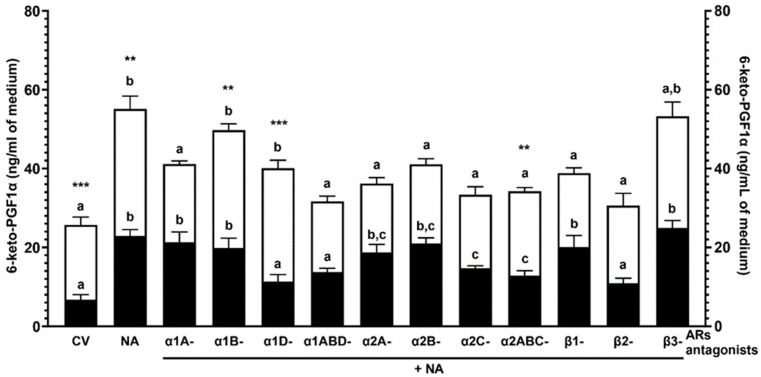
The influence of noradrenaline (NA, 10^−5^ M) alone or with antagonists of α1-, α2- and β-adrenoreceptors (ARs, 10^−4^ M) on the 6-keto-PGF1α (prostaglandin I2 metabolite) concentration in the medium after incubation of the endometria of pigs of the CON (black bars) and *E. coli* (white bars) groups, determined by ELISA. N = 5 for each group. Treatments were conducted in triplicate for each pig (n = 5) in the particular groups. Data are presented as the mean ± sem. Different letters (a, b, c) show statistical differences (*p* < 0.05–0.001) within each group for each subtype of α1- or α2-AR antagonists with NA or each subtype of β-AR antagonists with NA versus CV (control value, obtained from the CON or *E. coli* groups’ endometrial explants that had not undergone any treatment in vitro) and NA action alone; means marked by the same letter within each group show no significant difference; ** *p* < 0.01, *** *p* < 0.001 show statistical differences between groups for the same treatment.

The higher (*p* < 0.001) 6-keto-PGF1α content in the medium was detected after incubation of the endometria of both groups with NA versus the control values (Figure 2). In the *E. coli* group tissue, the control value (*p* < 0.001) and the response to NA (*p* < 0.01) increased versus the CON group.

#### 2.2.3. α1-AR Antagonists with NA

In the CON group endometria, α1A- and α1B-AR antagonists with NA increased (*p* < 0.001) the 6-keto-PGF1α medium accumulation compared to the control value (Figure 2). The metabolite content in response to α1D- (*p* < 0.01) and α1ABD (*p* < 0.05)-AR antagonists with NA was reduced compared to the NA influence. Following the exposure of the *E. coli* group tissue to α1B- (*p* < 0.01) and α1D (*p* < 0.05)-AR antagonists with NA, the 6-keto-PGF1α content increased versus the control value. The α1A- and 1ABD-AR antagonists with NA resulted in lower (*p* < 0.001) 6-keto-PGF1α content than the NA action. The α1B- (*p* < 0.01) and α1D (*p* < 0.001)-AR antagonists with NA increased metabolite content in the *E. coli* group in reference to the CON group.

#### 2.2.4. α2-AR Antagonists with NA

In the CON group, the use of α2A-, α2B- (*p* < 0.001), α2C- and α2ABC (*p* < 0.05)-AR antagonists with NA elevated the 6-keto-PGF1α medium accumulation versus the control value (Figure 2). The α2C- and α2ABC-AR antagonists with NA decreased (*p* < 0.05) the metabolite contents versus the NA action. In the *E. coli* group endometria, α2A-, α2B-, α2C- and α2ABC-AR antagonists with NA led to lower (*p* < 0.001) 6-keto-PGF1α content in the medium concerning the NA action. After the *E. coli* group tissue treatment with α2ABC-AR antagonist with NA, the contents of the metabolite increased (*p* < 0.01) compared to the CON group.

#### 2.2.5. β-AR Antagonists with NA

In the CON group, the 6-keto-PGF1α accumulation was increased by β1- (*p* < 0.01) and β3 (P<0.001) -AR antagonists with NA in reference to the control value (Figure 2). The content of this metabolite was lower (*p* < 0.05) in response to the β2-AR antagonist with NA versus the NA effect. After treatment of the *E. coli* group endometria with β1- and β2-AR antagonists with NA, the content of this metabolite decreased (*p* < 0.01) versus the NA action.

The statistical comparison between the action of particular antagonists of α1- or α2-AR subtypes/isoforms with NA or particular antagonists of β-AR subtypes with NA and the action of NA on the PGI2 metabolite concentration is shown in Figure 2.

### 2.3. Experiment 2.1: The Effect of NA and/or AR Agonists and Antagonists on PGIS Protein Abundance in LPS-Treated Endometrial Epithelial Cells

#### 2.3.1. AR Agonists and Antagonists Alone

In the studied cells, the PGIS protein abundance in response to α1- and α2-AR agonists and β1-, β2- and β3-AR antagonists was lower (*p* < 0.01) versus the NA action (Table 2). The β-AR agonist increased (*p* < 0.001) PGIS abundance in relation to the control value and the actions of α1- and α2-AR agonists and β1-, β2- and β3-AR antagonists.

#### 2.3.2. LPS Alone or with NA, AR Agonists and Antagonists

**Figure 3:**
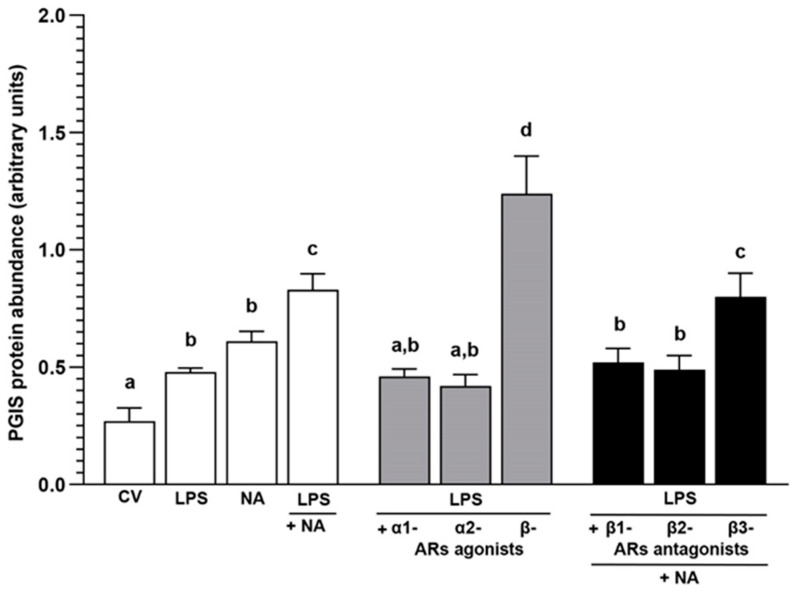
The influence of noradrenaline (NA, 10^−5^ M) alone or agonists of α1-, α2- and β-adrenoreceptors (ARs, 10^−4^ M) or antagonists of β-ARs (10^−4^ M) with NA on prostaglandin I synthase (PGIS) protein abundance in lipopolysaccharide (LPS, 10 ng/mL of medium)-treated porcine endometrial epithelial cells, determined by Western blotting. Treatments were conducted in triplicate for each pig (n = 4). PGIS values were normalised against glyceraldehyde-3-phosphate dehydrogenase (GAPDH) protein abundance. Data are presented as the mean ± sem. Representative bands for each treatment are presented on the blot (Figure S2). Different letters (a, b, c, d) show statistical differences (*p* < 0.05–0.001) between treatments. Means marked by the same letter show no significant difference. CV: control value.

In the cells, LPS and NA increased (*p* < 0.05) the PGIS protein abundance in reference to the control value (Figure 3). The PGIS abundance in the LPS-treated cells in response to NA was higher than the control value (*p* < 0.001) and LPS (*p* < 0.001) and NA (*p* < 0.05) actions. The α1- and α2-AR agonists in the LPS-treated cells led to a drop (*p* < 0.001) in PGIS abundance versus the action of LPS with NA. The β-AR agonist enhanced (*p* < 0.001) the enzyme abundance in reference to the control values and the effects of LPS, NA, LPS with NA, and LPS with α1- and α2-AR agonists. After using β1- and β2-AR antagonists with NA, the PGIS abundance in the LPS-treated cells was higher than the control value (*p* < 0.01) and lower (*p* < 0.01) versus the LPS with NA action. The β3-AR antagonist and NA increased PGIS abundance compared to the control value and the actions of LPS, NA (*p* < 0.001), and LPS with β1- (*p* < 0.05) and β2 (*p* < 0.01)-AR antagonists with NA.

### 2.4. Experiment 2.2: The Effect of NA and/or AR Agonists and Antagonists on the 6-keto-PGF1α Concentration in the Medium after Incubation of LPS-Treated Endometrial Epithelial Cells

#### 2.4.1. AR Agonists and Antagonists Alone

After using the β-AR agonist, the 6-keto-PGF1α medium concentrations were elevated (*p* < 0.001) in relation to the control value and the actions of NA, α1- and α2-AR agonists and β1-, β2- and β3-AR antagonists (Table 2).

#### 2.4.2. LPS Alone or with NA, AR Agonists and Antagonists

**Figure 4:**
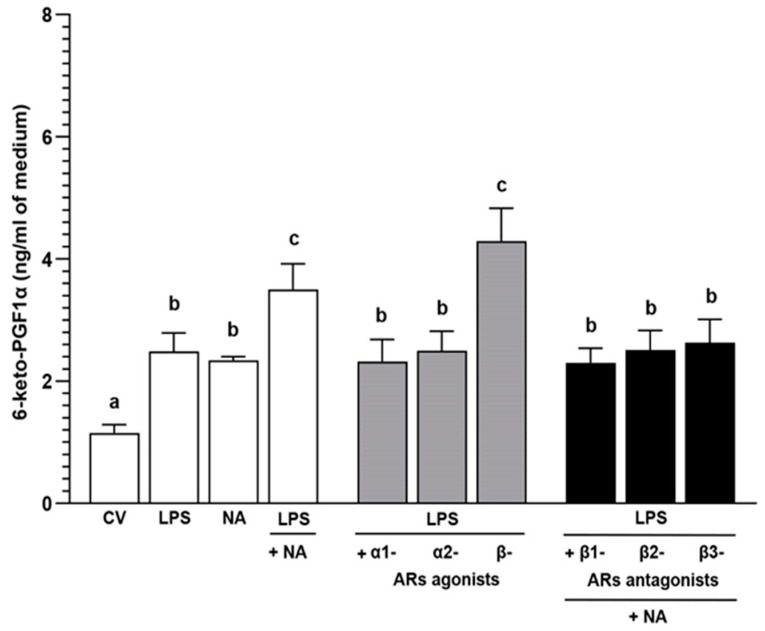
The influence of noradrenaline (NA, 10^−5^ M) alone or agonists of α1-, α2- and β-ARs (10^−4^ M) or antagonists of β-adrenoreceptors (ARs, 10^−4^ M) with NA on the 6-keto-PGF1α concentration (prostaglandin I2 metabolite) in the medium after incubation of lipopolysaccharide (LPS, 10 ng/mL medium)-treated porcine endometrial epithelial cells, determined by ELISA. Treatments were conducted in triplicate for each pig (n = 4). Data are presented as the mean ± sem. Different letters (a, b, c) show statistical differences (*p* < 0.05–0.001) between treatments. Means marked by the same letter show no significant difference. CV: control value.

The 6-keto-PGF1α medium content after incubation of the epithelial cells with LPS and NA increased (*p* < 0.001) versus the control value (Figure 4). In the LPS-treated cells, NA enhanced this metabolite content in reference to the control value (*p* < 0.001) and the LPS and NA influences (*p* < 0.05). After exposure to α1- (*p* < 0.01) and α2 (*p* < 0.05)-AR agonists, the 6-keto-PGF1α content in the LPS-treated cells medium was lower than the LPS with NA action. The β-AR agonist increased (*p* < 0.001) the metabolite content versus the control values and the actions of LPS, NA, and LPS with α1- and α2-AR agonists. To β1-, β2- and β3-AR antagonists with NA, the cells responded by the increase (*p* < 0.01) in 6-keto-PGF1α content in relation to the control value and by the drop (*p* < 0.05) versus the LPS with NA action.

## 3. Discussion

The involvement of neuronal regulation, including noradrenergic regulation, in PG formation in the inflamed uterus is poorly understood. For this reason, the present report investigated whether NA and ARs contribute to the control of PGI2 synthesis and release from porcine endometrial explants and epithelial cells under inflammatory conditions.

It is known that inflammation in the endometrium is maintained by interactions between factors released by the different cells within the endometrium, including immune, epithelial, stromal and endothelial cells [6,28,29]. To reflect the in vivo situation as much as possible, inflamed endometrial explants were used in the first part of the current study to determine the NA effect on PGI2 synthesis and release. After intrauterine injections with *E. coli* suspension, an inflammatory exudate was present in the horns, and the endometrium was red and swollen. In the endometrium, a severe acute inflammatory process was diagnosed based on the presence of the following changes: oedema, hyperaemia, damage to the luminal and glandular epithelium and a statistically higher number of neutrophils than in the healthy uterus [23]. It was observed that NA increased PGI2 release from the endometrial explants of pigs from the CON group, which coincided with an enhancement in PGIS protein abundance. To date, only the current findings determine the role of catecholamines in PGI2 formation in a healthy uterus. There are data in the literature showing the stimulatory action of NA and adrenaline on PGI2 release from the human myometrium [24]. Under physiological conditions, NA also increased PGI2 release from the rabbit [30] and rat [31] aorta and rat and cat venous [32] and arterial [33] tissue slices, as well as from the rat pulmonary ring [34]. Thus, the obtained findings confirm and deepen the understanding of the links between catecholamines and PGI2 in the uterus under physiological conditions. In the *E. coli* endometrium not treated in vitro, PGI2 release and PGIS protein abundance were enhanced versus the CON group, confirming previous results [10]. Completely new data are concerned with the NA-stimulated PGI2 synthesis and release from the inflammatory-changed endometrium. Similar to the healthy endometrium of pigs (CON group), NA augmented this PG release from the endometrium with inflammation, and it was greater compared to the healthy endometrium. In the tissue with inflammation, the higher PGI2 release by NA was convergent with the increased PGIS protein abundance, and it was enhanced versus the CON group. It is worth mentioning that in the endometrial explants with inflammation used in the present study, NA augmented the PG-endoperoxidase synthase-2 (PTGS-2) protein abundance [27]. Thus, it is hypothesised that an NA-enhanced increase in PGI2 synthesis in the endometrium with inflammation may result from increased PGIS and PTGS-2 expression.

In this study, antagonists of ARs were used to indicate the significance of particular ARs in PGI2 synthesis and release by endometrial explants and epithelial cells. The cells were also exposed to AR agonists. The antagonists alone did not significantly change PGIS abundance and PGI2 release by the explants of both studied groups in reference to the control values. PGIS protein abundance and PGI2 release were increased by NA alone versus the control values as well as those after the application of the action of the AR antagonists alone. The same actions were found in the epithelial cells in relation to both studied parameters after applying the antagonists and NA alone versus the control values. The β-AR agonist used in the study stimulated PGIS abundance and PGI2 release compared to the control values, as well as PGI2 secretion in reference to the NA action. After treatment with α1- and α2-AR agonists, PGIS abundance and PGI2 release were similar to the control values and decreased versus the NA influence alone. These findings agree with those found in the bovine endometrial [35] and the rabbit gastric epithelial [36] cells.

To date, studies on the role of ARs in the action of catecholamines on PGI2 release by a healthy uterus had only revealed that NA, as well as adrenaline, elevated this PG secretion by the myometrium of women acting by α- and β-ARs [24]. The current study supports the above findings, indicating the involvement of α1-, α2- and β2-AR subtypes in NA-stimulated PGI2 secretion by the endometrial explants from the CON group. Moreover, these ARs participate in the rise of PGIS protein abundance evoked by NA. To the best of the authors’ knowledge, the current study showed, for the first time, that α1D- and α2C-AR isoforms participate in the NA influence on PGI2 release from endometrial explants. Furthermore, these isoforms are important for NA action on PGIS protein abundance in the healthy endometrium.

In the endometrial explants from the *E. coli* group, the importance of α1- and α2-AR subtypes in the NA stimulatory influence on PGIS protein abundance and PGI2 release was found. Moreover, it was revealed that α1A-, α2B-, α2C-, β1- and β2-ARs participate in the NA stimulatory action on PGIS protein abundance, as well as in PGI2 release. NA, via α2-ARs, influences the release of PGI2 by inflamed endometrial explants. Between the examined groups, partial differences were revealed in the involvement of individual kinds of ARs in the NA action on the studied parameters. Contrary to the endometria of the CON group, in the *E. coli* group, α1A-, α2B- and β1-ARs mediate the NA effect on PGIS abundance and PG release. Similarly, the NA activation of α2A-ARs in inflamed endometrial explants increased PGI2 release. This may result from the augmented α2A- and β1-AR protein and mRNA abundance, respectively, revealed earlier in the inflamed endometrium presented in the current report. However, despite similar α1A- and α2B-AR mRNA and protein abundance in the CON and *E. coli* groups’ endometria [23], they are involved in the induction by NA of PGIS abundance and PGI2 release in the *E. coli* group, which may be a consequence of the different localisation of ARs and their different affinities for ligands. Further studies are needed to clarify this issue.

To better understand the cellular mechanisms of noradrenergic influence on PGI2 formation during endometritis, *E. coli* LPS-treated porcine endometrial epithelial cells were used in the second part of the study. The basis of this research is the presence of ARs and PGIS in these cells under physiological and inflammatory conditions [10,23]. Moreover, porcine endometrial epithelial cells, along with arterial cells, are the main site of PGI2 synthesis during inflammation [10]. Earlier, it was reported that *E. coli* LPS increased the release of PGF2α and PGE2 from bovine endometrial epithelial cells, but it was not accompanied by significant changes in the PGFS, microsomal or cytosolic PGES protein levels [4]. The NA stimulatory action on PGF2α release by bovine endometrial cells [35] and PGI2 secretion by rabbit gastric epithelial [36] and vascular smooth muscle cells [30], as well as by the homogenates of superior cervical ganglia [37], is also known. In the current study, porcine endometrial epithelial cells responded to LPS and NA with an elevation in PGIS protein abundance and PGI2 release. It should be stressed that LPS, together with NA, increased the values of these parameters more than LPS and NA alone. Similarly, NA enhanced PTGS-2 expression and PGE2 production induced by LPS in primary rat microglial cells [38]. The present findings show that the non-selective β-AR agonist stimulated PGIS protein abundance and PGI2 release from LPS-treated porcine endometrial epithelial cells. Further, the selected β1- and β2-AR antagonists (but not the β3-AR antagonist) blocked the NA stimulatory influence on PGIS abundance in the examined cells. In turn, the selective antagonists of β1-, β2- and β3-ARs resulted in a drop in NA-induced PGI2 release from the LPS-treated cells. This indicates that the effect of NA on PGI2 synthesis in LPS-treated endometrial epithelial cells is mediated via β1- and β2-ARs, while β1-, β2- and β3-ARs are involved in PGI2 secretion. The β-ARs, including subtype 2, participate in the NA action on PGF2α release by bovine endometrial cells [35], while β1- and β2-ARs mediate the NA-enhanced effect on PTGS-2 production in primary rat microglial cells [38]. In these cells, a lack of involvement of α-ARs was also reported [35,38]. The data confirm that α1- and α2-ARs did not contribute to PGI2 synthesis and release by *E. coli* LPS-treated porcine endometrial epithelial cells. However, the expression of α(1,2)-ARs occurs in the luminal and glandular epithelium of healthy and inflamed endometria in pigs [23].

As mentioned earlier, inflammatory uterus conditions led to the increase in the content of PGI2 metabolite in cow peripheral blood [13,14] and the porcine endometrium, myometrium and uterine washings. These changes in pigs were accompanied by stronger immunoreactivity for PGI receptors in the part of the luminal and glandular epithelium and the endometrial blood vessels [10]. Taking into account the local PGI2 influence, it should be stressed that this PG is a significant mediator of the inflammatory reaction. Together with PGE2, it induces changes in vascular permeability associated with hyperaemia and oedema during acute inflammation, and PGI2 elicits a nociceptive pain response [11,12]. PGI2 also exerts stimulatory action on the contractility of the inflamed porcine uterus [10]. By showing the role of NA and particular ARs in PGI2 formation and release by the porcine endometrium, including epithelial cells, under inflammatory conditions, the current report expands knowledge on the neurogenic control mechanism of PG synthesis and release during uterine inflammation. The knowledge obtained can be used to develop drugs (antagonists and/or agonists of particular AR isoforms/subtypes) to influence the course of the inflammatory reaction and uterine function.

## 4. Materials and Methods

### 4.1. Experiment 1: Aimed to Determine the Effects of NA and/or AR Antagonists on PGIS Protein Abundance and 6-keto-PGF1α Concentration in Endometrial Explants with E. coli-Induced Inflammation In Vivo

#### 4.1.1. Animals, Study Design and Collection of Uteri

The Local Ethics Committee for Experiments on Animals (University of Warmia and Mazury in Olsztyn, Poland) approved all study procedures (Consent no. 65/2015). The guidelines in EU Directive 2010/63/EU for animal experiments were also followed. During the experiment, special attention was paid to minimising the stress reaction in animals. They were also under constant (daily) veterinary care.

The experiment was performed on sexually mature gilts (Large White × Landrace) aged 7–8 months and weighing 107.3 ± 1.8 kg/mean ± sem. To detect behavioural estrus, a tester boar was used. In the experimental pigs, reproductive deviations were not revealed (vaginal discharges did not take place, and the second oestrous cycle occurred regularly). For acclimatisation, the gilts were transported from a commercial farm (Agro-Wronie Sp. z o.o., Wronie, Wąbrzeźno, Poland) to a local animal house (University of Warmia and Mazury, Olsztyn, Poland) three days prior to the start of the study. Each animal was housed in an individual pen (an area of about 5 m^2^). Gilts were kept at a temperature of 18 ± 2 °C, and the light conditions were as follows: natural daylight—14.5 ± 1.5 h, night—9.5 ± 1.5 h. The gilts were given a commercial diet and had access to water.

Following the acclimatisation period, on day 3 of the second oestrous cycle (day 0 of the study), the gilts were randomly divided into the CON group (n = 5, the gilts with saline injections into the uterine horns) and the *E. coli* group (n = 5, the gilts with *E. coli* suspension injections into the uterine horns). The research procedures were published earlier [39]. Briefly, premedication in gilts was induced by atropine (Atropinum sulf. WZF, Warszawskie Zakłady Farmaceutyczne Polfa S.A., Warsaw, Poland), azaperone (Stresnil, Janssen Pharmaceutica, Beerse, Belgium) and ketamine hydrochloride (Ketamina, Biowet, Puławy, Poland), and general anaesthesia was induced by ketamine hydrochloride. After the median laparotomy was performed, in the *E. coli* group, *E. coli* suspension (50 mL, content: 10^9^ colony-forming units (CFU)/mL, strain with a serotype O25:K23/a/:H1; Department of Microbiology, National Veterinary Research Institute, Puławy, Poland) was injected into each uterine horn. The choice of bacteria quantity was based on a previous report, which revealed that the intrauterine injections of the same strain of *E. coli* in the same amount as in the present study led to the development of severe (most frequently) or moderate acute endometritis after eight days [10]. In the gilts of the CON group, a saline solution (50 mL) was injected into each uterine horn. The horn of the uterus was injected with a bacterial suspension and saline solution in five places at a similar distance from each other, and 10 mL of bacterial suspension/saline was administered to each site. The animals from both groups were untreated in the period from surgery until euthanasia. The euthanasia of gilts was performed by an overdose of sodium pentobarbital on day 8 of the study (the expected day 11 of the oestrous cycle). The collected uteri were transported on ice to the laboratory (within 20 min).

#### 4.1.2. Preparation and Treatment of Endometrial Explants with NA and/or AR Antagonists

The uterine horns were washed twice using sterile phosphate-buffered saline (PBS, 137 mM NaCl/cat. no. 79412116, POCH, Gliwice, Poland/, 27 mM KCl/cat. no. 739740114, POCH, Gliwice, Poland/, 10 mM Na_2_HPO_4_/cat. no. 117992300, CHEMPUR, Piekary Śląskie, Poland/, 2 mM KH_2_PO_4_/cat. no. 742020112, POCH, Gliwice, Poland/; pH 7.4). A fragment of the wall was then harvested from the middle part of each uterine horn. The wall was divided into endometrial and myometrial layers with the help of a scalpel blade. The endometrial fragments were cut into slices weighing 60–70 mg, and then rinsed in Medium 199 (cat. no. M2520, Sigma, St. Louis, MO, USA). Single endometrial explants were put into glass vials with 2 mL of Medium 199, which contained 0.1% bovine serum albumin (BSA; cat. no. A2058, Sigma) and antibiotics (500 μL/500 mL gentamicin, 100 μL/500 mL neomycin, cat. no. G1272, N1142, respectively, both from Sigma). Preincubation and incubation of slices were performed in a shaking water bath (temperature: 37 °C, a humidified atmosphere: 95% air and 5% CO_2_). Preincubation was carried out for 1.5 h. The endometrial explants from both groups were then treated for 16 h, with fresh medium (control values) or exposed to NA alone (10^−5^ M, Levonor, Warszawskie Zakłady Farmaceutyczne Polfa, Warsaw, Poland) and AR antagonists alone (each antagonist at a dose of 10^−4^ M). The antagonists were used for α1A- (RS 17053 hydrochloride, cat. no. 0985), α1B- (Rec 15/2615 dihydrochloride, cat. no. 3284), α1D- (BMY 7378 dihydrochloride, cat. no. 1006) and α1ABD (doxazosin mesylate, cat. no. 2964)-ARs, α2A- (BRL 44408 maleate, cat. no. 1133), α2B- (ARC 239 dihydrochloride, cat. no. 0928), α2C- (spiroxatrine, cat. no. 0631) and α2ABC (yohimbine hydrochloride, cat. no. 1127)-ARs, β1- (RS-atenolol, cat. no. 0387), β2- (ICI 118551 hydrochloride, cat. no. 0821) and β3 (SR 59230A hydrochloride, cat. no. 1511)-ARs. The endometrial slices were also exposed to NA (10^−5^ M) with particular antagonists (each at a dose of 10^−4^ M). The antagonists were provided by Tocris Bioscience. Initial dilutions of the antagonists were made in accordance with the manufacturer’s instructions (α1D-, α2A- α2B-, α2C-, β1- and β2-AR antagonists were diluted in 0.2 mm-filtrated distilled water; α1A-, α1B-, α1ABD-, α2ABC- and β3-AR antagonists were diluted in dimethyl sulfoxide (cat. no. W387509, Sigma), and then placed at −20 °C. The final antagonist and NA solutions were prepared using the same medium as for preincubation and incubation of endometrial explants. Each treatment was performed in triplicate (five separate studies for particular groups, n = 5). PGF2α secretion after exposure to a nitric oxide (NO) donor (NONOate; at a dose of 10^−4^ M, cat. no. 82150, Cayman Chemical Co., Ann Arbor, MI, USA) was applied to control the reactivity of endometrial explants. The doses of NA, antagonists, and NONOate, as well as the time of incubation, were based on the findings from pilot experiments and in accordance with the authors’ earlier studies. Following incubation, the tissue explants were blotted with a paper filter, weighed and frozen at −80 °C until determination of PGIS protein abundance. The incubation medium was collected into tubes containing a solution of 5% EDTA (cat. no. 118798103, CHEMPUR, Piekary Śląskie, Poland) and 1% acetylsalicylic acid (cat. no. 107140422, POCH, Gliwice, Poland) (pH 7.4) and placed at −20 °C for 6-keto-PGF1α content estimation.

### 4.2. Experiment 2: Aimed to Determine the Effects of NA and/or AR Agonists and Antagonists on PGIS Protein Abundance and 6-keto-PGF1α Medium Concentration in E. coli LPS-Treated Endometrial Epithelial Cells

#### 4.2.1. Animals and Uteri Collection

Uteri from gilts (n = 4) were obtained from a local abattoir. The entire uteri were removed within 5 min after the gilts were slaughtered. A macroscopic examination of the ovaries was conducted to identify the phase of the oestrous cycle [40]. Uteri obtained on day 8 of the oestrous cycle were used because the cells isolated on this day easily became confluent in culture conditions. The uteri were kept on ice and immediately transported to the laboratory.

#### 4.2.2. Isolation of Endometrial Epithelial Cells

**Figure 5:**
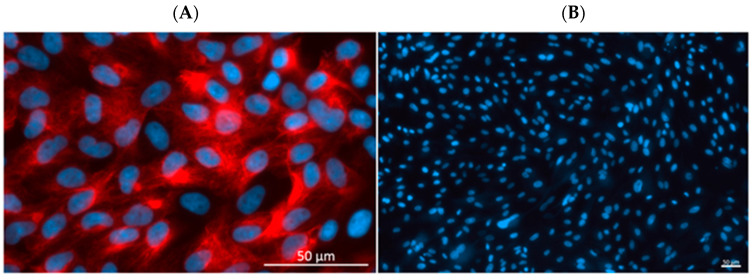
The purity of porcine endometrial epithelial cells was confirmed by immunofluorescent staining with antibodies anti-cytokeratin (**A**—positive control), with the typical pattern of an intermediate filaments clearly visible under x63 objective, and anti-vimentin (**B**—negative control). Scale bar: 50 µm.

Epithelial cell isolation from the endometrium was performed following the methodology described earlier [41] with modifications. Using sterile PBS supplemented with antibiotics (100 IU/mL penicillin and 100 µg/mL streptomycin; cat. no. 15140-122, Life Technologies, Bleiswijk, The Netherlands), horns of uteri were washed twice. The mid-portions of uterine horns were opened longitudinally on the mesometrial surface, and the endometrium was separated from the myometrium with scissors. The separation of these layers was confirmed under the dissecting microscope, and the endometrium was then cut into small slices. The endometrium was digested with 0.2% (*w*/*v*) dispase (cat. no. 17105041, Life Technologies, Grand Island, NY, USA) in Dulbecco’s PBS (cat. no. D5773, Sigma) at 37 °C for 50 min with gentle shaking. The obtained cell suspension was filtered through a 270 µm mesh to separate the remaining fragments of tissue. The released epithelial cells were resuspended with Medium 199 with 5% normal calf serum (NCS; cat. no. N4637, Sigma), pelleted by centrifugation at 200× *g* for 10 min and washed once with Medium 199 (cat. no. M5017, Sigma) containing 5% (*w*/*v*) NCS and antibiotics. Red blood cell lysing buffer (cat. no. R7757, Sigma) was applied to remove red blood cells from cell suspensions. The epithelial cells were subsequently rinsed three times with fresh Medium 199 containing 5% NCS and filtered through a 100 µm cell strainer (Becton Dickinson, Franklin Lakes, NJ, USA), and the fraction that passed through it was collected. The obtained cells were counted in a hemocytometer and seeded onto 75 cm^3^ culture flasks (2 × 10^6^ cells per 1 mL of medium). Cells were incubated at 37 °C in a humidified atmosphere of 95% air and 5% CO_2_ for 5 h. After this period, purified non-attached epithelial cells were collected, centrifuged, suspended in fresh Medium M199 with 10% NCS and seeded onto the new culture flasks. Cell viability was approximately 90%, as determined by the exclusion of 0.5% (*w*/*v*) trypan blue dye (cat. no. T6146, Sigma). The immunofluorescent staining pattern for the presence or absence of vimentin and cytokeratin [42] (using antibodies from Abcam, Cambridge, UK, cat. no. ab20346 and Sigma, cat. no. C1801, respectively) provided evidence that epithelial cell purity was between 85% and 90% (Figure 5). During the period in which cell cultures were conducted, the medium was replaced once every 24 h. Prior to the initiation of the experiment, the confluence of cells was assessed under a Zeiss Axioplan light microscope (Carl Zeiss, Oberkochen, Germany) and reached 80% to 90% after 5 to 6 days of culture.

#### 4.2.3. Treatment of Endometrial Epithelial Cells with LPS and/or NA, AR Agonists, Antagonists

Before exogenous factors were used, the epithelial cells were rinsed with fresh Medium 199 and then treated for 24 h with Medium 199 containing 2% BSA, 10% NCS and antibiotics containing no exogenous factors (control value) or with the addition of *E. coli* (O55:B5), LPS alone (10 ng/mL, cat. no. L2880, Sigma), NA alone (the same as for endometrial explants, 10^−5^ M), AR agonists alone (each at a dose of 10^−4^ M) for α1- (/R/-/-/-phenylephrine hydrochloride, cat. no. P6126), α2- (clonidine hydrochloride, cat. no. C7897) and β- (isoprenaline hydrochloride, cat. no. I5627), as well as AR antagonists alone (the same as for endometrial explants, each at a dose of 10^−4^ M) for β1-, β2- and β3-ARs. The epithelial cells were also treated with LPS (10 ng/mL) together with α1-, α2- or β-AR agonists (each at a dose of 10^−4^ M) or together with β1-, β2- or β3-AR antagonists (each at a dose of 10^−4^ M) and NA (10^−5^ M). All agonists were obtained from Sigma. Initial dilutions of the LPS and α1-, α2- and β-AR agonists were prepared according to the manufacturer’s instructions (in 0.2 mm-filtrated distilled water) and then stored at −20 °C. Initial dilutions of the used antagonists were prepared in accordance with the manufacturer’s instructions (the same as for endometrial explants) and then placed at −20 °C. The final agent solutions and the NA solution were prepared using the same medium as for cell culture. The release of PGF2α under the influence of NONOate (the same as for endometrial explants, at a dose of 10^−4^ M) was studied to control the reactivity of endometrial epithelial cells. The doses of the factors used and the time of incubation were based on the findings of pilot research or according to the authors’ previous studies. All treatments were conducted in triplicate, using cells isolated from four separate gilts. The media were subsequently harvested into tubes with a 5 µL EDTA and 1% acetylsalicylic acid solution (the same as for endometrial explants) and kept at −20 °C until determination of 6-keto-PGF1α concentration. The cells were allotted to determine PGIS protein abundance.

### 4.3. Western Blot Analysis

For homogenisation of the endometrial explants, a buffer with the following composition was used: 50 mM Tris HCl (pH 8.0), 150 mM NaCl, 1 mM EDTA, 1% (*v*/*v*) TritonX-100, 10 mg/mL aprotinin, 52 mM leupeptin, 1 mM pepstatin A, and 1 mM phenylmethylsulfonyl fluoride. The obtained homogenates were then centrifuged (10 min at 800× *g* at 4 °C). The supernatant was centrifuged for 1 h at 100,000× *g* at 4 °C to obtain the precipitate (applied as a microsomal fraction for the estimation of protein abundance of PGIS). This fraction was placed at −80 °C for further analysis. After harvesting the incubation medium, the endometrial epithelial cells were rinsed with PBS and lysed with 240 µL of ice-cold RIPA buffer (mmol/L Tris HCl, pH 7.4/cat. no. T150350, Sigma/; 150 mmol/L NaCl/cat. no. 794121116, POCH, Gliwice, Poland/; 1% Triton X-100 (*v*/*v*)/cat. no. T8787, Sigma/; 0.5% sodium deoxycholate (*w*/*v*)/cat. no. D6750, Sigma/; 0.1% sodium dodecyl sulphate (*w*/*v*)/SDS; cat. no. L3771, Sigma/; 1 mmol/L EDTA/cat. no. 879810429, POCH, Gliwice, Poland) together with protease inhibitor cocktail (cat. no. P8340, Sigma) and centrifuged for 5 min at 800× *g*. The supernatant was maintained at −80 °C for further analysis.

The total protein concentration was then determined [43]. Protein isolates (20 μg) from endometrial and epithelial cells were diluted in sodium dodecyl sulphate (SDS, cat. no. L3771, Sigma), a gel-loading buffer, heated (95 °C, 4 min) and separated by 10% SDS-polyacrylamide gel electrophoresis. The separated proteins were then electroblotted onto Immobilon^®^-P PVDF membranes (0.45 µm pore size, cat. no. IOVH00010, Sigma) in a buffer for transfer. To block nonspecific binding sites, the incubation was carried out with 5% fat-free dry milk (Spółdzielnia Mleczarska, Gostyń, Poland) in a Tris (cat. no. T1503, Sigma)-buffered saline containing Tween 20 (cat. no. P1379, Sigma) buffer (1.5 h, 21 °C). Afterwards, the membranes were exposed (18 h, 4 °C) to PGIS polyclonal rabbit antibody (in dilution 1:200; cat. no. 160640, Cayman Chemical Co.). The membranes were then placed under the influence (1.5 h, 21 °C) of a secondary antibody: alkaline phosphatase-conjugated goat anti-rabbit for PGIS (in dilution 1:10,000; cat. no. A3687, Sigma). Protein immune complexes were visualised by applying the standard alkaline phosphatase method (NBT-BCIP; cat. no. 72091, Sigma). Analyses were performed in triplicate. Earlier, the specificity of the PGIS antibody was reported in the gilt uterus [10]. The membranes were reprobed with polyclonal rabbit anti-glyceraldehyde-3-phosphate dehydrogenase (GAPDH) antibody (in dilution 1:5000; cat. no. G9545, Sigma). Images were acquired and quantified using a CHEMIDOC Touch Imaging System (Image Lab 5.2, Bio-Rad Laboratories, Hercules, CA, USA).

### 4.4. ELISA Procedure

The 6-keto-PGF1α concentration in the medium was measured using the ELISA kit (cat. no. 515211, Cayman Chemical Co.), following the manufacturer’s protocol. The standard curve ranged from 1.6 to 1000 pg/mL, and the detection limit was 6 pg/mL. The intra- and interassay coefficients of variation were 4.2% and 8.8%, respectively.

### 4.5. Statistical Analysis

The results obtained from the incubation of endometrial explants/cells were only considered if the release of PGF2α under the influence of NONOate was statistically significant. For explants, a two-way (group, treatment) ANOVA was used, and for cells, a one-way ANOVA followed by the Bonferroni test (InStat Graph Pad, San Diego, CA, USA) was applied to compare the mean (±sem) values. Differences with *p* < 0.05 were considered significant.

## 5. Conclusions

The current study showed that in the porcine endometrium with *E. coli*-induced inflammation, α1A-, α2(B,C)- and β(1,2)-ARs mediate the NA stimulatory action on PGIS protein abundance and PGI2 release, and the α2A-ARs are also involved in this PG release. In *E. coli* LPS-treated porcine endometrial epithelial cells, β(1,2)-ARs are activated by NA to increase PGIS protein abundance and PGI2 release. Additionally, β3-ARs participate in PGI2 release. In the course of spontaneous endometritis and metritis evoked by *E. coli*, these ARs may affect the ability of catecholamines to form and secrete PGI2. The results suggest that NA may indirectly affect the processes regulated by PGI2 (inflammatory process, uterine functions) in the inflamed uterus in animals and women. The results will contribute to a better understanding of the etiopathogenesis of uterine inflammation and may lead to the development of new strategies for prophylaxis and the treatment of uterine diseases to improve reproductive system function and the profitability of animal production.

## Figures and Tables

**Table 1:** The influence of noradrenaline (NA, 10^−5^ M) alone or antagonists of α1-, α2- and β-adrenoreceptors (ARs, 10^−4^ M) alone on the prostaglandin I synthase (PGIS) protein abundance in the endometrium and the 6-keto-PGF1α (prostaglandin I2 metabolite) concentration in the incubation medium of pigs of the CON and *E. coli* groups. N = 5 for each group. PGIS protein abundance was estimated by Western blotting, and values were normalised against glyceraldehyde-3-phosphate dehydrogenase (GAPDH) protein abundance. 6-keto-PGF1α concentrations were estimated by ELISA. Treatments were conducted in triplicate for the five pigs in particular groups. Data are presented as the mean ± sem. CV: control value (obtained from the CON or *E. coli* groups’ endometrial explants that had not undergone any treatment in vitro); anta.: antagonist.

Treatment	Group	PGIS Protein Abundance	6-keto-PGF1α Concentration
		(Arbitrary Units)	(ng/mL of Medium)
CV	CON	2.07 ± 0.28 ^a^	6.77 ± 1.26 ^a^
	*E. coli*	3.87 ± 0.34 ^a,^***	18.92 ± 1.98 ^a,^***
NA	CON	4.37± 0.11 ^b^	22.92 ± 1.58 ^b^
	*E. coli*	5.83 ± 0.31 ^b,^**	32.21 ± 3.24 ^b,^**
α1A anta.	CON	1.74 ± 0.09 ^a^	5.99 ± 0.26 ^a^
	*E. coli*	3.84 ± 0.12 ^a,^***	15.99 ± 0.21 ^a,^***
α1B anta.	CON	1.93 ± 0.22 ^a^	7.59 ± 0.31 ^a^
	*E. coli*	3.22 ± 0.33 ^a,^**	20.57 ± 0.26 ^a,^***
α1D anta.	CON	1.95 ± 0.52 ^a^	4.89 ± 0.12 ^a^
	*E. coli*	3.13 ± 0.13 ^a,^*	19.67 ± 0.19 ^a,^***
α1ABD anta.	CON	2.25 ± 0.03 ^a^	8.11 ± 0.21 ^a^
	*E. coli*	3.53 ± 0.09 ^a,^*	21.19 ± 0.31 ^a,^***
α2A anta.	CON	2.25 ± 0.23 ^a^	6.94 ± 0.19 ^a^
	*E. coli*	3.39 ± 0.14 ^a,^*	15.93 ± 0.29 ^a,^***
α2B anta.	CON	1.95 ± 0.14 ^a^	7.55 ± 0.26 ^a^
	*E. coli*	3.54 ± 0.41 ^a,^***	18.47 ± 0.38 ^a,^***
α2C anta.	CON	2.22 ± 0.23 ^a^	8.14 ± 0.31 ^a^
	*E. coli*	3.47 ± 0.34 ^a,^**	21.52 ± 0.34 ^a,^***
α2ABC anta.	CON	2.21 ± 0.12 ^a^	9.64 ± 0.38 ^a^
	*E. coli*	3.31 ± 0.08 ^a,^*	17.85 ± 0.41 ^a,^***
β1 anta.	CON	2.29 ± 0.56 ^a^	4.69 ± 0.25 ^a^
	*E. coli*	3.52 ± 0.13 ^a,^**	16.98 ± 0.39 ^a,^***
β2 anta.	CON	2.42 ± 0.04 ^a^	7.87 ± 0.2 ^a^
	*E. coli*	3.31 ± 0.07 ^a^	20.35 ± 0.32 ^a,^***
β3 anta.	CON	1.84 ± 0.06 ^a^	6.69 ± 0.26 ^a^
	*E. coli*	3.44 ± 0.09 ^a,^***	18.48 ± 0.35 ^a,^***

Different letters (a, b) show statistical differences (*p* < 0.001) for α1- or α2-AR subtype/isoform antagonists or β-AR subtype antagonists in relation to CV and NA influence for PGIS or 6-keto-PGF1α within each group; means marked by the same letter within each group show no significant difference; * *p* < 0.05, ** *p* < 0.01, *** *p* < 0.001 show statistical differences between groups for the same treatment.

**Table 2:** The influence of noradrenaline (NA, 10^−5^ M) alone or agonists of α1-, α2- and β-adrenoreceptors (ARs, 10^−4^ M) alone or antagonists of β-ARs (10^−4^ M) alone on prostaglandin I synthase (PGIS) protein abundance in porcine endometrial epithelial cells and the 6-keto-PGF1α concentration (prostaglandin I2 metabolite) in the incubation medium of cultured endometrial epithelial cells without LPS. PGIS protein abundance was determined by Western blotting, and values were normalised against glyceraldehyde-3-phosphate dehydrogenase (GAPDH) protein abundance. 6-keto-PGF1α concentrations were determined by ELISA. Treatments were conducted in triplicate for each pig (n = 4). Data are presented as the mean ± sem. CV: control value; agon.: agonist; anta.: antagonist.

Treatment	PGIS Protein Abundance	6-keto-PGF1α Concentration
	(Arbitrary Units)	(ng/mL of Medium)
CV	0.27 ± 0.03 ^a^	1.15 ± 007 ^a^
NA	0.48 ± 0.01 ^b,c^	2.34 ± 0.03 ^b^
α1 agon.	0.26 ± 0.05 ^a^	1.47 ± 0.09 ^a^
α2 agon.	0.22 ± 0.04 ^a^	1.23 ± 0.11 ^a^
β agon.	0.65 ± 0.03 ^c^	3.65 ± 0.07 ^c^
β1 anta.	0.25 ± 0.04 ^a^	1.41 ± 0.05 ^a^
β2 anta.	0.23 ± 0.05 ^a^	1.26 ± 0.13 ^a^
β3 anta.	0.21 ± 0.03 ^a^	1.32 ± 0.09 ^a^

Different letters (a, b, c) show statistical differences (*p* < 0.05–0.001) for PGIS or 6-keto-PGF1α. Means marked by the same letter show no significant difference.

## Data Availability

All relevant data are contained within the manuscript.

## Supplementary Materials

The following supporting information can be downloaded at: https://www.mdpi.com/article/10.3390/ijms25126313/s1.
