# Supplementary material for: Noradrenaline and Adrenoreceptors Are Involved in the Regulation of Prostaglandin I2 Production in the Porcine Endometrium after Experimentally Induced Inflammation
Source: Int J Mol Sci. 2024 Jun 7;25(12):6313. doi: 10.3390/ijms25126313 (PMC11204358; doi:10.3390/ijms25126313)
Supplement: Supplementary file 1 [file ijms-25-06313-s001.zip › ijms-2941940-supplementary.pdf]

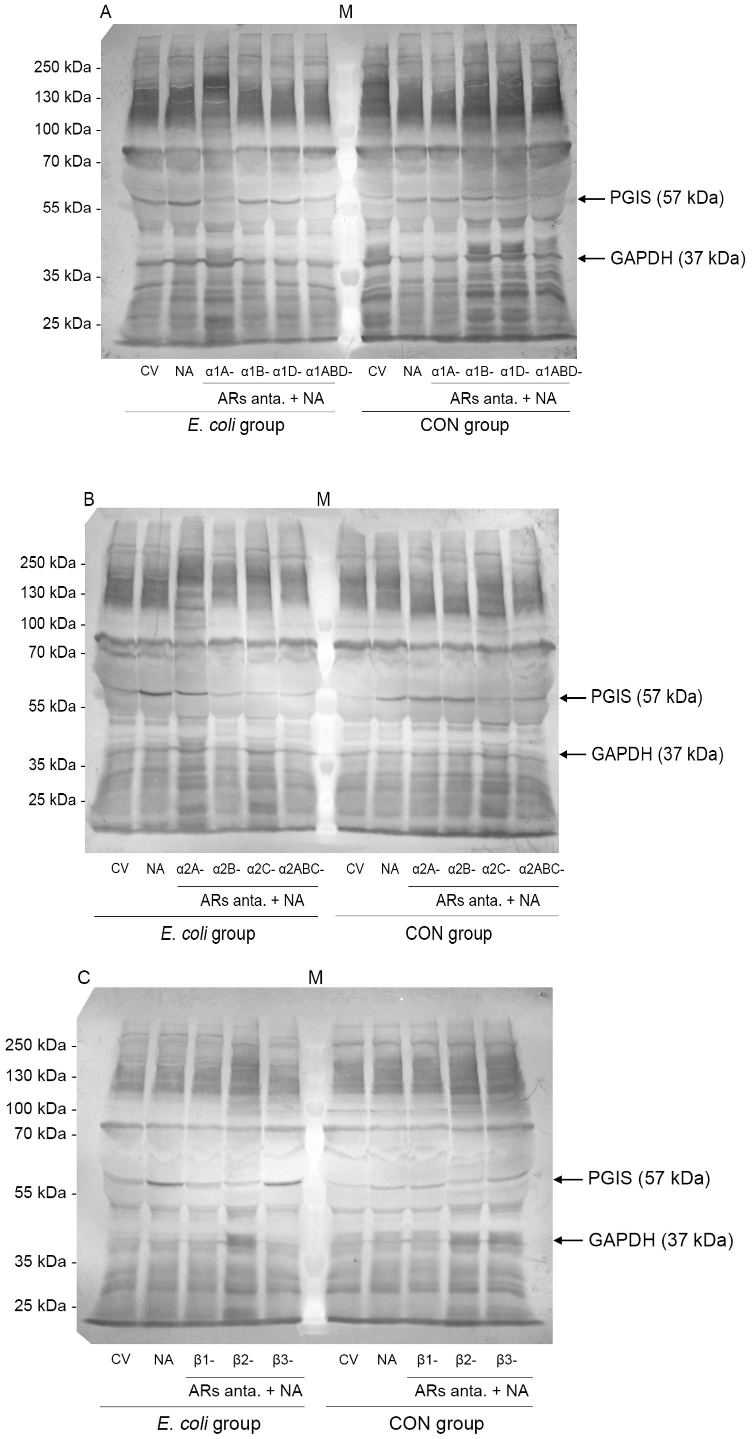

Supplementary Figure S1. The influence of noradrenaline (NA) alone or with antagonists of α1- A), α2- (B) and β (C) -adrenoreceptors (ARs) on the prostaglandin I synthase (PGIS) protein abundance in the endometrium of pigs from *E. coli* (*E. coli*) and control (CON) groups, determined by Western blotting. The density of bands was normalized in relation to glyceraldehyde-3-phosphate dehydrogenase (GAPDH). For PGIS antibody bands are visible at 57 kDa, for GAPDH antibody at 37 kDa. M: marker; CV: control value (obtained from the CON or *E. coli* groups endometrial explants that had not undergone any treatment *in vitro)*; anta.: antagonist.

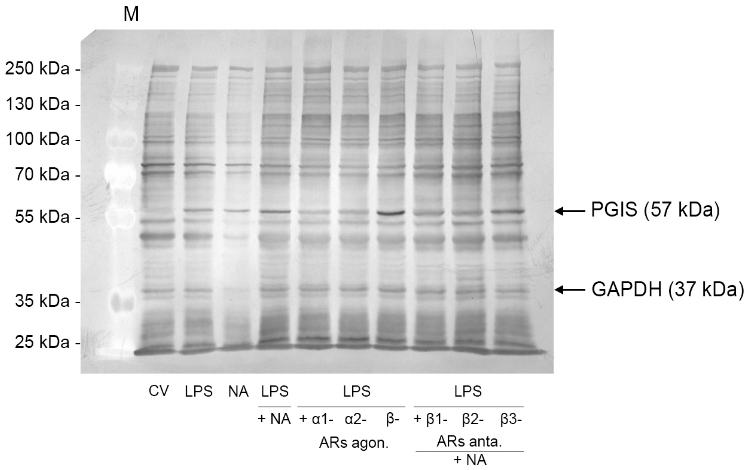

Supplementary Figure S2. The influence of noradrenaline (NA) alone or agonists of α1-, α2- and β-adrenoreceptors (ARs) or antagonists of β-ARs with NA on the prostaglandin I synthase (PGIS) protein abundance in the lipopolysaccharide (LPS)-treated porcine endometrial epithelial cells, determined by Western blotting. The density of bands was normalized in relation to glyceraldehyde-3-phosphate dehydrogenase (GAPDH). For PGIS antibody bands are visible at 57 kDa, for GAPDH antibody at 37 kDa. marker: M: marker; CV: control value; agon.: agonist; anta.: antagonist
